# Minimally invasive colorectal cancer procedures in patients with obesity: an interdisciplinary approach

**DOI:** 10.1007/s10151-019-02027-5

**Published:** 2019-07-04

**Authors:** K. Alyaqout, A. Lairy, E. Efthymiou, H. Khwaja, O. Warren, S. Mills, P. Tekkis, C. Kontovounisios

**Affiliations:** 1 Department of Colorectal Surgery, Chelsea & Westminster Hospital, London, UK; 2 Department of Bariatric Surgery, Chelsea & Westminster Hospital, London, UK; 3 Department of Colorectal Surgery, Royal Marsden Hospital, London, UK; 4 Department of Surgery and Cancer, Imperial College London, Chelsea and Westminster and the Royal Marsden Campus, London, UK

## Introduction

The obesity rate has tripled since 1975, and over 650 million adults were obese in 2016 [1]. Obesity is an overwhelming yet relatively neglected health problem [2]. In the United Kingdom overweight and obesity were the underlying causes of 11% of colorectal cancer (CRC) [3].

Minimally invasive colorectal cancer procedures (MICCP) are gaining popularity. The rise in the rates of cancer, obesity and minimally invasive surgery will increase the demand for elective MICCP. Yet, although this approach is attracting many surgeons, it carries an array of challenges in individuals with obesity.

A multimodal approach to manage this multifactorial problem is imperative; moreover, it should begin early in the patient’s therapeutic journey. This article highlights solutions to overcome some of the technical challenges seen in MICCP. Bariatric and colorectal surgeons in our center collaborated in an attempt to improve the surgical outcome in obese patients undergoing elective CRC procedures, with special attention to elective MICCP (Table 1).

**Table 1:** Summary of recommendations

Type of cancer/stage	BMI	Peri operative		Preoperative						Intraoperative	Post-operative		
		MIS recommended	Include in clinical pathway	Waiting restriction	Nutrition assessment	VLCD	VLCD duration	VLCD Type	Risk prediction scores	Optical trocar/ICG/Long instruments/bariatric theatre standards and tables	Revise dosing regimen for all drugs	OSA and HDU considerations	Surveillance
Colon													
Stage I	≥ 30 k/m^2^	Yes	Yes	No	CT body composition	If no sarcopenia	1–2 weeks		Yes	Yes	Yes	Yes	Yes
Stage II	≥ 30 k/m^2^	Not in T4	Yes	No	CT body composition	If no sarcopenia ± in open	1–2 weeks		Yes	Yes	Yes	Yes	Yes
Stage III	≥ 30 k/m^2^	Not in T4	Yes	No	CT body composition	If no sarcopenia ± in open	1–2 weeks		Yes	Yes	Yes	Yes	Yes
Stage IV	≥ 30 k/m^2^	Not in T4 Only if respectable liver and/or lung metastasis	Yes if operable?	No if operable	CT body composition	If no sarcopenia ± in open	1–2 weeks		Yes	Yes	Yes	Yes	Yes
Rectum													
Stage I	≥ 30 k/m^2^	No unless experienced in Lap. TME or high-risk tumours (i.e., positive margin)	Yes	Yes	CT body composition	If no sarcopenia ± in open	1–2 weeks		Yes	Yes	Yes	Yes	Yes
Stage II	≥ 30 k/m^2^	No unless experienced in Lap. TME or high-risk tumours (i.e., positive margin	Yes	Yes	CT body composition	If no sarcopenia ± in open	1–2 weeks		Yes	Yes	Yes	Yes	Yes
Stage III	≥ 30 k/m^2^	Not in T4 No unless experienced in Lap. TME or high-risk tumours (i.e., positive margin	Yes	Yes	CT body composition	If no sarcopenia ± in open	1–2 weeks		Yes	Yes	Yes	Yes	Yes
Stage IV	≥ 30 k/m^2^	Not T4? If respectable lung and/or liver metastasis post chemoradiotherapy	Yes If operable	Yes if operable	CT body composition	If no sarcopenia ± in open	1–2 weeks		Yes	Yes	Yes	Yes	Yes

*MIS* minimally invasive surgery, *TME* total mesorectal excision, *CT* computed tomography, *VLCD* very low calorie diet

## Perioperative considerations

### Standardization of care

The standard enhanced recovery after surgery adopted by colorectal surgeons lacks specifications for patients with obesity. A perioperative pathway for obese patients (PPOP) has proven to reduce adverse events. Boodaie et al. showed significant reduction in the return-to-theatre unplanned readmissions rates and length of hospital stay. They have also shown that utilization of a morbid obesity-specific antibiotic protocol, which avoids underdosing, reduces the rate of superficial and deep infections. Such protocols may also reduce operating time by avoiding delays through standardising equipment and instruments [4]. In theory, PPOPs will help produce tailored chemotherapy and venous thromboembolism (VTE) prophylaxis protocols. They may also allow the introduction of bariatric CRC multidisciplinary meetings and the concept of intraoperative collaboration between bariatric and colorectal surgeons.

## Preoperative considerations

### Waiting time

A longer waiting time from diagnosis to surgery has no effect on disease-specific survival in colon cancer. On the contrary, preoperative optimization reduces postoperative complications, along with length of stay, and mortality [5]. Preoperative optimization in patients with obesity might include applying concepts such as preoperative weight reduction (POWR). A delay in waiting time was associated with a drop in overall survival in rectal cancer, hence the above cannot be recommended in obese patients with rectal cancer.

### Nutritional assessment and preoperative weight reduction

There is a misconception that all patients with obesity are well nourished. The truth is, a proportion of these patients suffer from sarcopenia [6]. None of the malnourishment screening tools used in oncology can accurately assess sarcopenia or myosteatosis. Recent innovations such as Body Composition Computed Tomography Assessment (BCCTA) can fill in this gap [7]. Determining the nutritional status is critical to determine if these patients are candidates for POWR.

When malnutrition is ruled out, preoperative diet (e.g., very low calorie diet), pharmacotherapy (e.g., liraglutide) or both can result in significant drop in the body mass index before surgery [8]. In MICCP reducing the adiposity of the mesentery and downsizing the liver can enhance the visibility and increase the intraabdominal space, thus allowing safer identification and dissection of critical structures and vessels. The exposure gained from a shrunken liver helps with colonic mobilization too. Furthermore, free intraabdominal volume increases, thus allowing better small bowel displacement cranially when needed. This may reduce the need for a steep head down position.

### Preoperative case and surgeon selection

The degree of operative difficulty is variable across patients with obesity. Male gender, central obesity, hepatomegaly, fatty liver, and super-obesity are clinical predictors of complex laparoscopic surgery. The learning curve should always be addressed, thus, risk prediction scores can be used to limit difficulties related to surgeons’ inexperience, in a case-difficulty/patient-experience matching fashion.

## Intraoperative considerations

Primary trocar entry can be challenging, therefore optical trocars are widely used by bariatric surgeons and could be adopted by colorectal surgeons. In MICCP, suboptimal dissection, mobilisation and vessel ligation may lead to prolonged operating times, poor resection or conversion to open. Indocyanine green (ICG) enhanced fluorescence is an evolving technology, with a relatively low cost and excellent safety profile. In MICCP, it has been used to assist in vessels’ and ureters’ identification by injecting ICG intravenously and in the bladder, respectively. This real-time angiography can help overcome the problem of vascular recognition in the heavy mesentery. Furthermore, better ureteric identification should reduce the risk of iatrogenic injury. ICG can also facilitate peritumoral lymphatic mapping in CRC. Optimizing laparoscopic ergonomics using long instruments is almost always necessary in patients with obesity. Maintaining proper manipulation and elevation angles (45°–75°, and 60°, respectively) may reduce surgeons’ exhaustion, which may require using a standing stool in some cases [9].

A“bariatric standard” intraoperative set up including anaesthetic theatre settings, suitable bed/trolley and operating table, gel padding, wide strapping, table extensions/arm boards, forearm cuff or large blood pressure cuff, ramping device—oxford pillow in theatre or beach chair position—can be adopted on the trolley. Steps for the anaesthetist, difficult airway equipment including a glidescope, ventilator capable of PEEP and pressure modes, a hover mattress, long spinal, regional and vascular needles, ultrasound machine, depth of anaesthesia monitoring, quantitative neuromuscular monitoring and nursing staff trained in both bariatric and CRC operations is needed.

## Postoperative considerations

The presence of bariatric service alone does not result in improvement in the postoperative outcome for obese patients with CRC. POPP should address various aspects of postoperative management such as, prophylaxis, treatment, and surveillance. Doses of antibiotics should be adjusted to avoid underdosing. Chemotherapy carries a number of controversies, especially that the American Society of Clinical Oncology Clinical Practice Guidelines recommend dosing according to actual weight, and warn that worse outcomes are seen in underdosing resulting from the unsupported fear of increased toxicity [10]. This may have also contributed to worse local control in rectal cancer for example and thus to the need fora different postoperative surveillance program for earlier detection.

## Conclusion

Collaboration between healthcare-workers is one of the pillars of the modern healthcare system. Treatment of obese patients with CRC can be the point where bariatric surgeons and their colorectal colleagues meet. Concepts such as dedicated PPOP and POWR may be the key to allow more access to MICCP. A PPOP serves many purposes and makes the tools required for better MIS experience consistent and readily available, it also helps with the pre-operative and postoperative optimisation for patients and limits the variables seen in management, allowing better understanding of where the pitfalls really are. POWR is widely used in bariatric surgery, and should be further investigated in CRC patients having MICPP and theoretically, it should help overcome some of the anatomical challenges seen in patients undergoing MIS for CRC. With the use of newer technologies such as ICG, the colorectal surgeon may feel more comfortable performing MICCP. Finally, limiting the challenges seen in obese patients with CRC is complex and thus requires a comprehensive multidisciplinary perioperative approach.
